# Designing a resilience model for pharmaceutical supply chain during crises: a grounded theory approach

**DOI:** 10.1186/s40545-021-00399-4

**Published:** 2021-12-30

**Authors:** Peivand Bastani, Omid Sadeghkhani, Ramin Ravangard, Rita Rezaei, Parisa Bikine, Gholamhossein Mehralian

**Affiliations:** 1 Health Human Resources Research Center, School of Management and Medical Informatics, Shiraz University of Medical Sciences, Shiraz, Iran; 2 Student Research Committee, Shiraz University of Medical Sciences, Shiraz, Iran; 3 School of Pharmacy, Shahid Beheshti University of Medical Sciences, Tehran, Iran

**Keywords:** Pharmaceutical supply chain, Resilience, Disaster, Grounded theory, Consumable medical equipment

## Abstract

**Background:**

During disasters or crises, the traditional models of supply chain encounter failure and skewedness under the inevitable and unknown pressures. The procurement and transformation of required equipment to the involved areas is considered as one of the main triggers of decreasing damages and losses during crisis. In this regard, a breakdown in pharmaceutical supply chain can lead to intensive, undesired consequences.

**Methods:**

This was a qualitative study applying a grounded theory approach. The study was conducted with attending of 32 informant participants who were qualified in supply chain during natural disasters and crisis. In order to collect the data, deep semi-structured interviews were applied along with investigating the documents, observation, field notes and theoretical memos. For data analysis, a continuous comparison was used according to Corbin and Strauss method.

**Results:**

Results of the study were categorized in 8 main categories as the main themes. “Wasting” appeared as the main factor of the resilience of pharmaceutical and consumable medical equipment supply chain. Wasting included two subthemes of loss of resources and wasting time.

**Conclusion:**

In order to make resilience in pharmaceutical and consumable medical equipment during disasters, it is necessary to reinforce the various dimensions of the resilience model to increase the rate of supply chain responsiveness. This study particularly contributes to broadening and deepening our understanding of how to mitigate the risk of undesirable outcomes of pharmaceutical supply chain during the disasters or crises.

## Background

The past few decades have been notable for major disruptions in supply chains [1]. It is estimated that in the first half of 2018, about 1069 disruption happened, which has been the highest rate in 3 years [2]. Catastrophic events such as the recent COVID-19 pandemic in 2020, hurricane Harvey in Houston in 2017, hurricane Maria in Puerto Rico in 2017, the 2010 eruption of a volcano in Iceland, and the 2011 Japanese tsunami caused profound damages to both private and public sectors [3]. Among them, pharmaceutical and medical device supply chain has been a very challenging issue worldwide and also has been one of the most prominent concerns of both policymakers and practitioners. The logic behind this concern is that these sort of products have to be provided at the right time with the reasonable price [4]. For this reason, all stakeholders try to do their best to supply such products with less defect. Obviously, this would be more highlighted in the unexpected situations where this supply chain is substantially influenced by external forces. Natural disasters are very common examples of these situations that has to be handled very fast; otherwise, it causes vast damages to both health status of a society and the environment.

At the time of disaster, the supply chain acts as a neural network, and traditional models of it fail to work properly because of the inevitable and unknown pressures. Failure in the supply chain may have an effect on all the product processes and lead to lose the capitals, increase the costs and reduce the market share along with customer dissatisfaction and brand damages. Tsunami and the earthquake in Japan along with the flood and nuclear event in Thailand have shown that how an event in a limited point of the world can damage half of the supply chains in the whole world. According to Tang (2006), supply chain risk management (SCRM) is ‘the management of supply chain risks through coordination or collaboration among the supply chain partners so as to ensure continuity’ [5]. It is largely argued that SCRM is intended to identify, assess, and monitor the risks for potential disruption in a supply chain continuum mitigating the negative consequence of disruptions on supply chain operations [6]. Supply chain risks are generally classified into disruption risks and operational risks [7]. As Mellat Parast and Shekarian (2020) note, disruption risks typically limit a supply chain system; they may originate man-made or natural events disasters such as hurricanes [8]. In this regard, in order to increase the resilience and flexibility against disasters, the organizations need to have new tools to assess the risk, supervise the supply chain as well as modeling [9]. It is important to remember that, this issue is wider than an organization’s role and requires a continuous improvement and supervision via related organizations in the supply chain [10]. The resilience of the supply chain not only leads to improve the efficiency and decrease the risk, but also causes to predict and moderate the inevitable events related to the supply chains quickly [9].

In this regard, pharmaceuticals are among vital necessities for patients all over the world and should be offered to them in all conditions as well as disasters and hazards [11]. According to the evidence, procurement of pharmaceuticals and transporting them to the damaged areas via supply chain is considered as one of the major issues of decreasing damages and injuries during disasters [12]. On the other hand, lack of pharmaceuticals is a huge problem in normalization of the disasters and those who leave their houses during crisis may have a severe need to treatment services and medicines as well [13]. At the same time, predicting the rate of pharmaceuticals consumption and the adequate ordering at the right time are considered as basic principles of health care [14].

While the procurement of consumable medical equipment and pharmaceuticals are among one of the vital priorities in developing countries [15], the challenge of scarcity in medical equipment and supply chain management in health scope can be more sensitive and with more fatal consequences [16]. It will be obvious that during disasters including a contagious outbreak, an industrial accident, a natural disaster, a man-made or terrorist occurrence, the importance of access to pharmaceuticals and consumable medical equipment becomes highlighted [17].

In sum of what was said, an efficient supply chain should be able to offer the pharmaceuticals in a rational manner and acceptable quality in a minimum possible time and the optimal costs [18]. Such this supply chain should have the potentiality of encountering the disasters and the flexibility of movement to the appropriate condition [19]. In this regard, some items can lead to the improvement and appropriate response of the pharmaceutical supply chain during disasters the same as: predicting the dynamic demand, flexible allocating of the resources, determining the shelf life of the consumable items, disseminating and sharing the information and developing the business models for minimizing corruption and fraud [17].

In our knowledge, most of the studies on supply chain in health care systems are focused on the normal conditions, and there are only limited numbers concentrated on health-related supply chain management during disasters. This study aims to propose a customized, resilient supply chain model for pharmaceuticals and consumable medical devices during disasters.

## Methods

### Study design

This was a qualitative study applying grounded theory approach with Strauss and Corbin’s philosophical orientation [20]. The aim of the study was to determine the process and the model of resilience in pharmaceuticals and consumable medical equipment supply chain during natural disasters. Strauss and Corbin’s philosophical orientation was applied rather than those of Glaser’s and Charmaz’s. Grounded theory has several distinct methodological categories: traditional GT associated with Glaser; evolved GT associated with Strauss, Corbin and Clarke; and constructivist GT associated with Charmaz. While there are commonalities among all generations of GT, there are factors that distinguish them such as the philosophical position of the researcher; the use of literature; and the approach to coding, and analysis and theory development. The evolved GT is founded on symbolic interactionism and stems from work associated with Strauss, Corbin and Clarke. Symbolic interactionism addresses the subjective meaning people place on objects, behaviors or events based on what they believe is true [21].

### Participants

The study population consists of those managers and staff affiliated in the organizations with the mission of pharmaceuticals and consumable medical equipment procurement and with the experience and presence of working in the natural disasters` scenes. 32 people were included in the study including 4 specialists with the degree of PhD, 9 medical doctors, 7 pharm doctors, 7 allied medicine experts and 5 participants with management education. The participants of the study first were selected purposefully among food and drug deputy of those Iranian Universities of Medical Sciences that were experienced any kinds of natural disasters the same as flood, earthquake, etc. After fulfilling 6 interviews and analyzing the achieved data, according to the theoretical sampling in grounded theory approach [22], data directed us to conduct interviews with the staff affiliated with Red Crescent Population, military organizations, insurance companies and distributors of pharmaceuticals and consumable medical equipment. In accordance with the lack of new codes in 5 latest interviews, the researchers received the saturation level and the sampling process was stopped. The average age of the participants were 43 years from the minimum of 29 to the maximum range of 54 years. The educational level of the participants was diverse from Bachelor degree to specialized PhD on different majors and specialized education in medicine and pharmacology and pharmacy.

In order to catch a better understanding of the resilience of pharmaceuticals and consumable medical equipment supply chain, we attempted to select the participants from a vast spectrum of those people who were engaged in recent national natural disasters the same as Bam earthquake (2003), East Azerbaijan earthquake (2012) and Kermanshah earthquake (2018) as well as Khorramabad flood (2019). In this regard, the first interview was conducted with a person who had an experience of attendance at the scene of disaster and then he was wanted to introduce the other informants with the similar experiences. So the sampling process was started purposefully and it was continued theoretically.

### Data collection

Deep and semi-structured interviews were applied. Written informed consent was obtained from all the participants and all the interview sessions were recorded. The interview protocol was prepared according to the aim of the studies and after conducting two pilot open interviews with two experts who were not mentioned as the main participants. After analyzing the content of the aforementioned pilot interviews, the initial draft of the interview protocol was developed and the meaningfulness and robustness of the questions were verified via two random interviews which were not included in the final analysis. The interview sessions were started with an open question: “what was your experience from pharmaceuticals and consumable medical equipment during disasters like?” then according to the participant’s descriptions, the probing questions were conducted. The interview protocol was then re-organized according to extracted categories from the previous interviews. The mean of the interview sessions was about 45 min and the duration of each interview session according to the condition and time of the participants was varied between 30 and 80 min. Each participant was interviewed between 1 or 2 times. Complementary interviews were conducted by phone and sometimes in a face to face condition in order to achieve more clarification and decrease the ambiguities. All the interviews were implemented during September 2018 to January 2020 in a period of 17 months.

### Data analysis

For data analysis, constant comparative method suggested by Strauss and Corbin was used including three stages of open coding, axis coding and selective coding [23]. For the open coding, first the recorded files of the interviews were changed to transcript and the whole body was reviewed several times and line by line then the main meaningful units and concepts in each line or paragraph were extracted and coded. The coding process was conducted applying the own words of the participants or the interpretations of the researchers from the original concepts extracted from the data. In this step, an initial categorization of the codes was made and 792 initial codes were extracted. Then after going through the constant comparative analysis, the initial codes were decreased to 209 final codes that finally were categorized in 30 subthemes and 8 main themes. The data analysis process was done applying MAX QDA—2010.

**Fig. 1:**
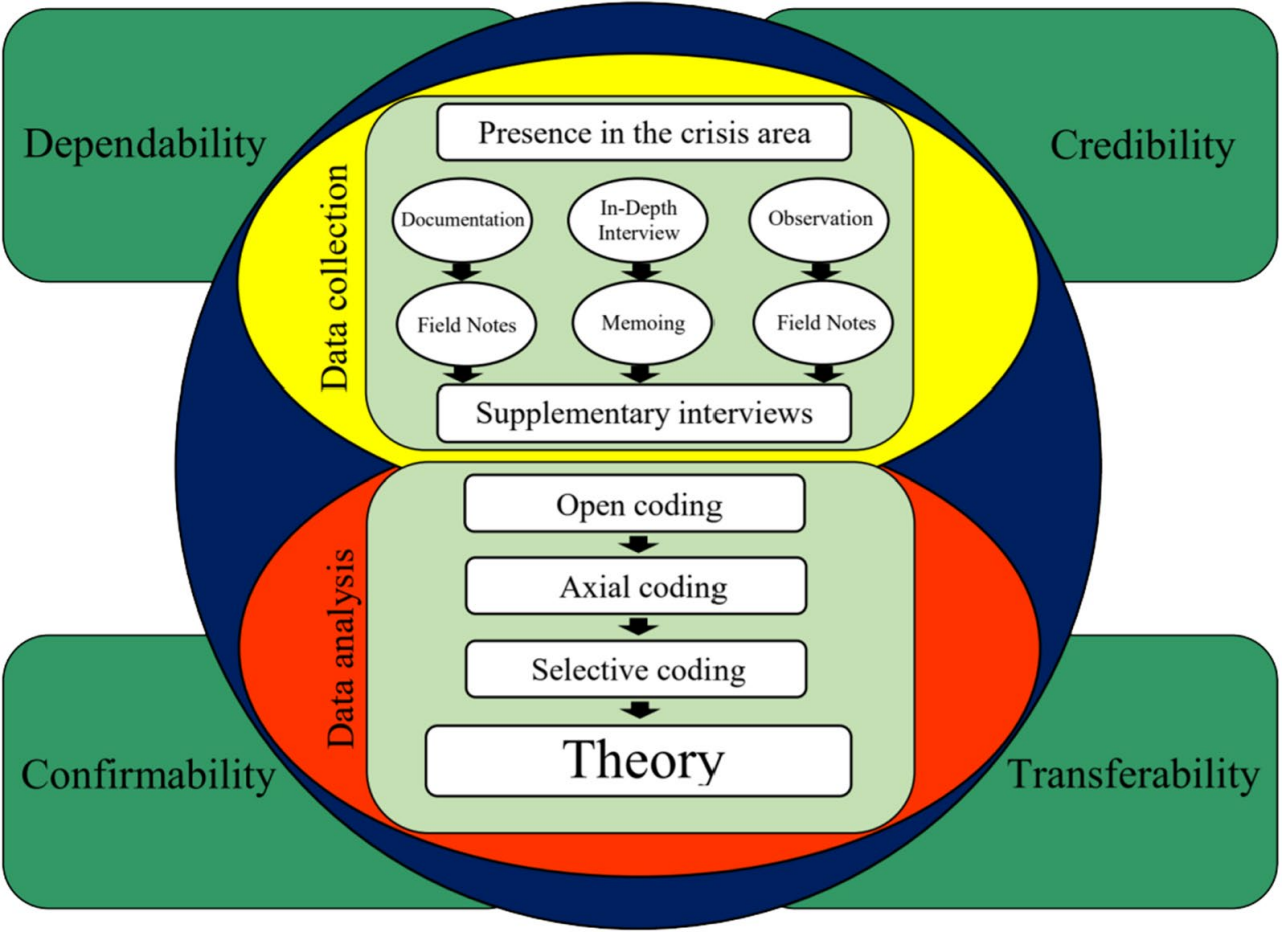
The research method process

In order to assure the rigor and validity of the data, four Lincoln and Guba’s criteria were applied including credibility, transferability, dependability and conformability [24]. Long-term involving of the researchers with the data led to achieve the credibility. Triangulation technique was another approach that was applied for better comprehending of the process and increasing data credibility. At the same time, using audio and video documents, field observations, filed notes and memos were applied. The sampling diversity, applying member check technique for reviewing the transcripts by the participants, peer reviewing the extracted codes by the colleagues and experts all over the research process and finally, external audit at the end of the research were among other techniques using for assuring conformability. Also, in order to achieve procedural precision, data management strategies and demonstrable procedural logic, the eight steps of developing grounded theory enhanced by Manuj et al. (2012) was applied as follows: explanation of the phenomenon, establishment of the appropriateness of the grounded theory methodology, selection of the context, selection of the data sources, development of the interview protocol, data collection and coding, diagraming concepts and developing the theory and comparing and evaluating the theory [25]. Figure 1 illustrates the research design framework, summarizing the processes used in the study.

## Results

**Fig. 2:**
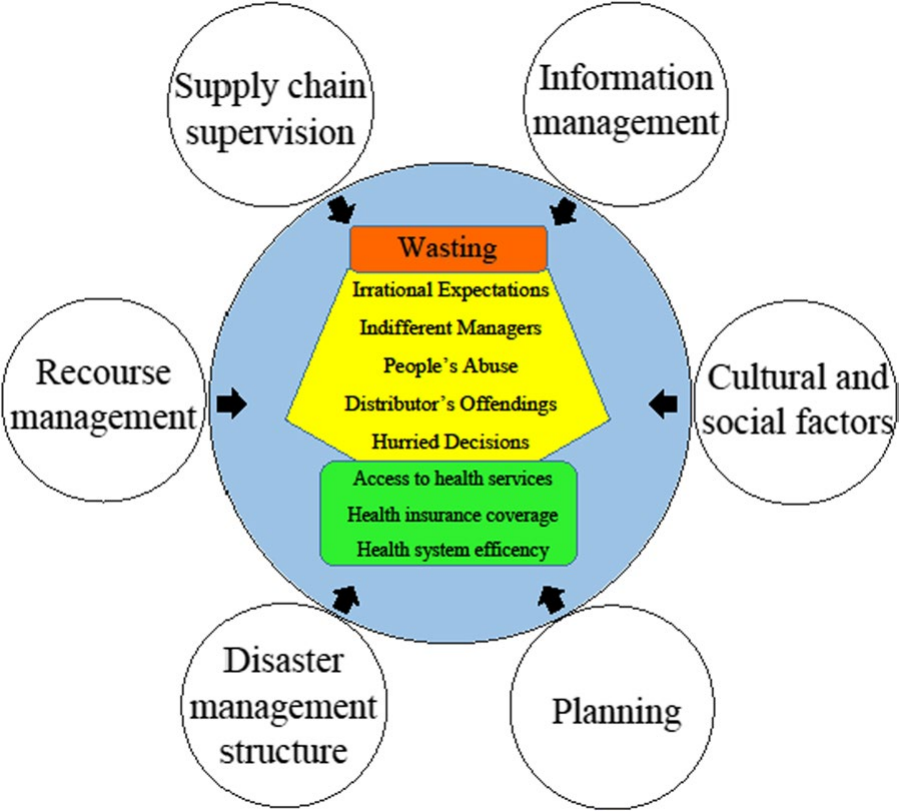
The conceptual model of resilience

Data analysis included 792 initial codes that after a continuous comparison of these codes and the raw data considering similarity and differences, the similar codes were categorized and labeled in a category. 209 categories were created and after integration they were summarized in 30 subthemes and 8 themes (Table 1). The present results have led to “wasting” of the pharmaceuticals and consumable medical equipment during natural disasters as the main phenomenon of the research (Fig. 2).

**Table 1:** The main themes and subthemes of resilience model

Main themes	Subthemes
Disaster management structure	Infrastructure standards
	Health system potentials
	Appropriate designing of the structures
	Supply chain stewardship
	Unique command and initial actions
Information management	Comprehensive information of the managers
	Appropriate need assessment and creating an integrated information system
Supply chain supervision	Distributors’ offending
	Controlling the probable outbreaks
	Controlling of the local condition and respecting safety principles
Sociocultural factors	Level of literacy
	Employment
	Age
	Religion
	Expectations and requests
Planning	Nonintegrated service delivery
	Importance of decision-making
	Necessity of planning
Resource management	Resource shortage
	Supply chain management
	Donation management
	Workforce management
Health service coverage	Supply chain restrictions
	Health insurance coverage
	Healthcare system efficiency
	Access to health cares
Wasting	Human resource wasting
	Financial wasting
	Wasting time

### Disaster management structure

Disaster management structure is considered as an important determinant in managing pharmaceuticals and consumable medical equipment supply chain during disasters. This main theme contains 5 subthemes of infrastructure standards, health system potentials, appropriate designing of the structures, supply chain stewardship, unique command and initial actions.

About the structure standards, one of the participants declared that:

*“If the health centers encountered the earthquake, they would not have enough consistency. In the experience of Sarpol-e Zahab earthquake those governmental buildings of the health centers had sufficient consistency against those of private or charity sectors that do not have so much solidity” [P*_*3*_*].*

About utilization of the health system potentialities, another participant added:

*“One strength point in our country is the existence of healthcare centers and hospitals with near distances, because of this, we always have amount of medicine and medical equipment even for the consumption of several months according to the condition and numbers of referees” [P*_*7*_*].*

Appropriate designing as another subtheme was mentioned here:

*“Right at the earthquake night a comprehensive team of medical university and drug and food deputy was attended as a disaster in governors’ office but the promptitude was not enough. It seems that the present structure does not have enough efficiency and should be redesigned” [P*_*23*_*].*

Kermanshah earthquake has implied the importance of unique command and stewardship of pharmaceutical and consumable medical equipment during disasters. In this regard, a participant said:

*“In early 24 h the needed structures were made and food and drug deputy was assigned as the steward of medicine and medical equipment’s distribution to the damaged areas” [P*_*17*_*].*

### Information management

Information in disasters can help to identify the local condition and problems in order to assess the needs and better management of supply chain. The related subthemes in this regard were: comprehensive information of the managers, appropriate need assessment and creating an integrated information system.

Comprehensive information of the managers considers very important during disasters and should be reached via training the managers with principles of disasters and consistency of the management. One participant declared that:

*“A person should be responsible of a supply chain that has enough experience and is aware of the process. Such a person can act more rapidly than those who do not have necessary experience and specialty” [P5].*

According to the present results, inefficient structure of need assessment was an obvious problem in Kermanshah earthquake. A participant said:

*“During disasters, food and drug deputy as an agent of Ministry of health should assess the pharmaceutical needs and allocate local and national resources to these requirements, but unfortunately this is not happened in reality” [P*_*7*_*].*

An integrated information system is among the most significant information management principles during disasters. A participant clarified:

*“There was lack of integrated information system and unique management. The only thing that we really required was a management unit that can integrate the information with online monitoring, assess daily needs and plan for them…” [P*_*12*_*].*

### Supply chain supervision

The need of supply chain supervision is obvious during disasters because of preventing probable abuse and readiness for encountering the contagious outbreaks. The related subthemes were as follows: pharmaceutical distributors’ offending, controlling the probable outbreaks, controlling of the local condition and respecting safety principles. Regarding the conflict of interest toward the pharmaceuticals and medical equipment suppliers and distributors, the possibility of corruption and offending during disasters is not unexpected. In this regard, one of the participants clarified his experience:

*“If we do not check the delivered packages, the distributors may send whatever they want to the damaged areas without considering our needs. This condition may include fake recipients of the items, sending expired medicines or those with only a short shelf life” [P*_*3*_*].*

Controlling the epidemics in damaged areas regarding the geographical and critical circumstances of the field should be sped up. One participant declared:

*“The needs may become different second by second according to the condition. In Sarpol-e- Zahab we have experienced epidemic crisis the same as prevalence of Cutaneous Leishmaniasis that needs extra requirements to be controlled the same as environmental interventions, poison spraying, etc.” [P*_*25*_*].*

Management and control of the local condition during crisis was among other important subthemes. One of the participants stated:

“According to the climate and seasonal needs, the condition became different. The requirements of the damaged areas were greatly related to the type of crisis, severity of the disaster, the volume of collapse, the population and geographical conditions. All these variables can affect the management of the supply chain*” [P*_*29*_*].*

Finally, health care workforces’ respect of the safety principles during service delivery was among other subthemes. This subtheme becomes more considerable knowing the psychological problems affect the personnel during disasters. One participant remembered his experiences as follows:

*“In such a condition many of the healthcare workers may attempt to do some think because of their empathy and emotional forces, in this regard, if a shortage is occurred or a new problem is emerged, they may forget about the safety procedures and try to help under any circumstances” [P*_*11*_*].*

### Cultural and social factors

One of the other determinant factors affecting the resilience of pharmaceutical and medical equipment supply chain is the sociocultural characteristics of the damaged areas that can affect the improvement of resource allocation and relief process or worsen the condition. According to the present results, level of literacy, employment, age, religion and the expectations and requests of the damaged people from the healthcare system are among the important sociocultural factors. In this regard, one of the participants added:

*“The cultural of the people in damaged areas is a determinant factor in disaster management. For example, if they have ever experienced similar condition or the level of expectations. Of course in rural and retrieved areas, it becomes manageable easier than the metropolitans with higher expectations of the citizens and their resistance to change” [P*_*19*_*].*

### Planning

Planning was a key determinant that was considered in all the interviews. The participants indicate lack of planning in pharmaceutical and consumable medical equipment supply chain. The related subthemes were: non-integrated service delivery, importance of decision-making and necessity of planning.

The participants have claimed that healthcare system may experience a shock caused by the disaster. This shock can affect the potential of resilience of the system and may lead to non-integration and disorder. One participant said:

*“In many disasters as well as Bam, Bushehr, Azerbaijan and Kermanshah earthquakes, we have faced anarchy particularly in 2–3 initial days. This chaos can lead to the system’s shock. So in this way we cannot have an integrated information system about the real needs, exact estimation of the damages and the affected population as well. All of these may cause lack of integrated services by different organizations during disasters*” [P_4_].

The necessity of urgent and significant decisions is among main characteristics of critical conditions. But the present results indicate the wrong, delayed and arbitrary decisions in many situations. One participant noted:

*“unfortunately we always postpone the decision-making to the disaster condition rather than predicting and preparing before that. So in such an emergency condition we are forced to decide promptly and in a hurry” [P*_*21*_*].*

The participants have indicated the inefficiency and inappropriateness of their organizations’ action plans for real crisis. One participant added:

*“we do not have a systematic national plan for disasters. We may conduct workshops and meetings but an executive plan is missing always. As a result, in disasters, the organizations act separately and according to their local instructions” [P*_*9*_*].*

### Resource management

The present results have emphasized the importance of financial and manpower resources during natural disasters. The main subthemes here are as follows: resource shortage, supply chain management, donation management and workforce management. According to the participants, the access to health services may decrease regarding the type and severity of the crisis and shortages may be highlighted. One participant exemplified:

*“pharmaceutical system among all other health care sections allocate a considerable amount of costs so during disasters, the consumption of medicines and consumable medical equipment may increase and the shortages will be occurred” [P*_*16*_*].*

The participants have mentioned supply chain management as a determinant factor in resource management during disasters. A participant affiliated in a military organization clarified:

“as an allied organization, we can help the healthcare organizations during disaster but our main responsibility is not health service delivery and we cannot procure all pharmaceuticals and medical equipment requirements*” [P*_*13*_*].*

Another finding was donations. Some part of these gifts delivered from all over the country was not practical and necessary and some of them were over the needs. A participant said:

*“many of the donated items were out of our control and cannot be organized. For example, 20 packages of gloves were sent that we did not really need. Many other items that were donated from all over the country also were not needed and we did not even have the condition for their storage” [P*_*20*_*].*

About another subtheme, human resource management through supply chain, we should point to two sections of specialists’ management and the management of the volunteers. A participant added:

*“If there was a documented plan for the actions along with the trained workforces, the problems could better be managed. We needed trained volunteers with adequate familiarity with the condition of damaged areas and their culture and even language” [P31].*

### Health service coverage

Health service coverage was divided to four related subthemes as follows: supply chain restrictions, health insurance coverage, healthcare system efficiency and access to health cares. According to the participants, supply chain restrictions included distributive restrictions and legal requirements. A participant said:

*“when we had 2 or 3 destinations for medicines, our logistic potentiality was decreased for appropriate distribution” [P13].*

Increasing the costs of services during disasters requires a rapid intervention of insurance organizations. In this regard, another participant added:

*“We had all the services freely in the first month after earthquake, but in the second month, we tried to relate the population needs with their insurance coverage. Many of the injured people had an insurance coverage but unfortunately their needs were not reimbursed by the insurers and the Ministry of Health had to pay” [P*_*8*_*].*

Inefficiency of the healthcare system was among another subtheme that was made because of increasing in load of services and unneeded referrals. A participant pointed out:

*“the volume of patients was great during the earthquake and the other provinces helped us a lot. At the same time, collapse of health infrastructures imposed a great load of actions to our governmental and even private centers. All the surgeons were on call 24 hours… all of these along with the emotional and psychological dimensions led to system’s inefficiency” [P11].*

Transportation problems also had led to barriers in physical access to pharmaceuticals and medical equipment supply chain. One participant stated his experience as follows:

*“that night when we were on a way to deliver medicines and medical equipment to Sarpol-e-Zahab, the landside was happened and the rural ways were chocked. This collapsed the process of relief and unfortunately we missed that vital hours” [P*_*10*_*].*

### Wasting

Wasting was the main and core determinant in the study. This was the most intellectual theme that joins all the other themes to each other. According to the participants, wasting includes all the waste and overuse that occurred in human resources, financial resources and time. In this regard, one of the participants emphasized on the wasting of financial and human resources as follows:

*“we had a good access to workforces and volunteers with high level of energy but unfortunately we could not utilize and manage these valuable sources” [P*_*5*_*].*

About financial wasting, another participant added:

*“although we have a rich country, we are not able and trained to save. In Kermanshah earthquake we really did not have serious resource shortages but the main problems were appeared in resource allocation” [P*_*17*_*].*

And finally, time wasting was among the other subthemes. A participant declared that:

*“we always act and decide in a hurry so we waist out main capital, time, if we were ready before disaster, we never behaved in a try and error way that caused wasting our golden time” [P*_*11*_*].*

## Discussion

This study was aimed to determine the model of resilience in the supply chain of pharmaceuticals and consumable medical devices during natural disasters. Wasting was the main determinant theme of the study that is caused following the ambiguity in the supply chain stewardship, lack of unit command, lack of integrated information system, awareness of the managers, and non-integrated and fragmented services during disaster. During crisis, supply chain can encounter various problems in structure of the disaster management, planning, infrastructure standards, and comprehensive need assessment.

Other studies indicate the same results for example, it has been indicated a severe lack of coordination and preparedness against disasters inter and among rehabilitative centers. Such a condition can be a result of neglecting the rule of preparedness guidelines and a definite structure as a main coordinator any crisis [26]. Dash et al. (2013) have proposed an interactive model of supply chain during disaster, including strategic planning, coordination, resource allocation, information seeking, communication, collaboration, transportation, monitoring, costs, time, performance assessment and documentation of lessons learned [27]. It would be obvious that only in the condition of a near collaboration among the stated factors, a supply chain can be reacted appropriately during crisis. Similarly, a previous study in Finland has declared that using local relief infrastructures are among the best way of overcoming the critical situations [28]. It is claimed that a dollar cost in preparedness and infrastructure designing is equivalent to 3 dollars in response to disaster stage [29].

The management of the supply chain`s information systems is significant for collaborating and sharing the information among internal and external customers, suppliers, distributors, and other stakeholders [30]. According to the present results, information diversity among different organizations, incomplete statistics and information, inappropriate information infrastructures during disasters are among the integrated information system’s problems. In this regard, some scholars have proposed a radio frequency identification (RFID) for inventory management and control and improvement of response system [31]. It is also claimed that RFID can be used for management and tracing of the packages and valuable products [32]. Some researchers have also stated that implementing of RFID can lead to optimized management of medicines and consumable medical devices inventories [33]. In order to achieve the highest level of the performance, supply chain strategies should be matched with the information systems’ strategies [34]. Although the values related to information technology are related to both technological and managerial skills, the size of an organization, the previous success, uncertainty, support of high management, and power of stakeholders may affect supply chains [35].

During disasters, the possibility of offending, fake documentations and any kind of corruptions becomes highlighted, which requires the strongest supervision on the pharmaceutical supply chain. Meanwhile, in order to control the condition of the damaged regions and estimate the need for effective interventions,, some necessary factors should be considered, among them we can point to isolation, environmental health, vaccination consistency and the necessity of psychological interventions. For instance, Rachaniotis et al. (2012) put more emphasis on the role of management of the supply chain and logistic operations to control the prevalence of epidemic outbreaks during disasters [36]. In addition, Gupta et al. have suggested a model of decision-making in selecting the best location for setting the health centers and implementing vaccinations during crisis [37].

As far as social and cultural determinants are concerned, life style of the damaged areas can highly affect the population`s expectations, and consequently, emotional reactions of the people and health care workers can make difficulties in service delivery during disasters. Hence, attention to the community health literacy and training the population to enable for encountering disasters via social media would be helpful. It seems that a long-term and systematic plan can lead to improving the social and cultural aspects of the population as well as education and appropriate correction interventions in the countries with the same setting like Iran. In addition, as indicated by Christopher and Jüttner (2000), it is so crucial to manage the relationship with other parts of supply chain to increase the efficiency the supply chain [38].

With respect to the importance of flexible and applied planning, a previous research has also emphasized on agile pharmaceutical chain that is forged by speed of delivery, planning, trust development, prioritization of the suppliers, and the performance assessment [39]. It is also concluded that natural disasters such as flood and earthquake can lead to the collapse of pharmaceutical supply, and identification of the vulnerable areas and implementing the pre-planned interventions were among the suitable solutions during the critical conditions [40].

Related to resource management, it is shown that the main reasons of low preparedness of hospital during disasters can be associated with human resources coordination and management [41]. Additionally, Kwon & Kim (2018) in south Korea have interpreted that any investing on preparedness stages is much more effective than costing for utilizing relief actions [42]. In a study conducted in China, it is demonstrated that an emergency supply chain needs to determine supply points, emergency logistic centers and demand points in order to respond quickly [43].

Access to health services and health centers, developing surge capacities and vast coverage of the damaged areas are among other results of the study. Insurance coverage can be a solution that should be considered to reach a comprehensive coverage of damaged populations. Along with these requirements, the legal restrictions of the supply chain should be considered. Deficiency in distribution chain among community as well as hospital pharmacies and the problems related to local distribution system should be considered as well. About the efficiency of the health care system, we also have to mention the high load of works in primary days right after the crisis and service delivery in a higher level than the personnel’s potential capacity. It is mentioned that nurses as a main group of engaged staff do not have the sufficient level of preparedness and some of them did not train for disaster conditions [44].

The reduction of the health workers’ productivity, useful application of volunteers and lack of attendance to the people’s creativity and experiences are among the main implications of human capital wasting in this study. Furthermore, increasing the pert of recourses during the supply chain and high costings for storage are among the implications of physical resource wasting in the present study. Delays in preventive actions before crisis occurrence, hurried actions and wrong decisions during crisis and delayed sending of recourses, duplications and unnecessary activities in health service delivery are among other findings that lead to time wasting. Reduction or elimination of these factors may lead to achieve an agile supply chain. Some scholars have emphasized that allocating enough funds for personnel compensation, investing enough time and energy for surveillance of the supply chain and dissemination of update and on-time information are among the important pre-disaster actions that can considerably decrease the time, money and energy wastes [45]. Finally, it is shown that an appropriate supply chain should mention the proportion between simplicity and complexity and achieving maximum output with minimum input to be able to respond to critical conditions [46].

## Conclusion

Results of the study have shown that in a resilience model of pharmaceutical and consumable medical devices supply chain during disasters, wasting of resources is being highlighted as a main determinant. Weak structure of health system disaster management, lack of planning at different levels and unclear information infrastructures may intensify the rate and scale of wasting during natural disasters. Increasing the rate of wasting in supply chain along with inappropriate expectations, indifferent managers, people’s probable abuse of resources, offending of pharmaceutical distributors and hurried decisions can lead to different outcomes the same as inefficiency of health system, reduction of health service access and health insurance coverage. In order to increase the resilience of pharmaceutical and consumable medical devices supply chain during disasters, reinforcing the present model’s dimensions should be taken for granted. Given the uncertainties and unexpected conditions that healthcare systems may face in the future, building on the grounded theory, this study provides them with a framework to deal with such situations in an efficient manner. In other words, the resilient model provided in this research contributes healthcare systems as to how they should proactively manage their limited resources and prevent the likelihood waste of resources.

### Research limitations and scope for future research

This study has some limitations similar to other qualitative studies. First, as the research findings are not empirically tested to discover if they are statistically significant, the findings cannot be generalized to wider populations with the same degree of certainty that quantitative studies can. Second, rare phenomena usually receive the same amount of importance as more frequent phenomena. As for theory evaluating, further research can be done to study how the findings can affect the resiliency of pharmaceutical and consumable medical devices supply chain.

## Data Availability

The datasets used and/or analyzed during the current study are available from the corresponding author on reasonable request.

## References

[1] Hosseini S, Ivanov D, Dolgui A (2020). Ripple effect modelling of supplier disruption: integrated Markov chain and dynamic Bayesian network approach. Int J Prod Res.

[2] Zhu Q, Krikke H, Caniëls MC (2017). Integrated supply chain risk management: a systematic review. Int J Logist Manag.

[3] Chen J, Xu H, Zhou P (2020). Delegation vs direct sourcing revisited: contract types under correlated supply risks and asymmetric cost information. Int J Prod Res.

[4] OECD. Guidelines for resilience systems analysis. OECD Publishing Paris; 2014.

[5] Tang CS (2006). Robust strategies for mitigating supply chain disruptions. Int J Log Res Appl.

[6] Kamalahmadi M, Shekarian M, Mellat PM (2021). The impact of flexibility and redundancy on improving supply chain resilience to disruptions. Int J Prod Res.

[7] Chen J, Sohal AS, Prajogo DI (2013). Supply chain operational risk mitigation: a collaborative approach. Int J Prod Res.

[8] Shekarian M, Mellat PM (2021). An Integrative approach to supply chain disruption risk and resilience management: a literature review. Int J Log Res Appl.

[9] Harrington L, Smith R. The resilient supply chain. DHL Supply Chain. 2014.

[10] Gary L. Supply chain resiliency: how prepared is your organization? : Marsh Inc.; 2012.

[11] Bahrin NLZ, Hassan Y, Abd Majeed AB, Zulkifli NW, Ahmad A. Pharmaceutical Fiscal Sustainability: Review of Malaysia’s Essential Medicines List. 2nd International Conference on Public Policy; Università Cattolica del Sacro Cuore, Milan, Italy 2015.

[12] Lin Y-H, Batta R, Rogerson A, Blatt A, Flanigan M. Logistic model for delivery of critical items in a disaster relief operation: heuristic approaches2009. 1–42 p.

[13] Ochi S, Hodgson S, Landeg O, Mayner L, Murray V (2015). Medication supply for people evacuated during disasters. J Evid Based Med.

[14] Barnett J (1980). Supply of medicines text book of hospital pharmacy.

[15] Hogerzeil HV (2006). Essential medicines and human rights: what can they learn from each other?. Bull World Health Organ.

[16] Pan American Health Organization. Natural disasters: protecting the public’s health. PAHO Washington; 2000.

[17] Graves S, Lei L, Melamed B, Pinedo M, Qi L, Shen Z, et al., editors. New challenges to emergency management of pharmaceutical/healthcare supply chain disruptions. DHS Workshop on Incident Management, Resource Management, and Supply Chain Management; 2009.

[18] Jaberidoost M, Nikfar S, Abdollahiasl A, Dinarvand R (2013). Pharmaceutical supply chain risks: a systematic review. DARU J Pharm Sci.

[19] Christopher M, Peck H (2004). Building the resilient supply chain. Int J Logist Manag.

[20] Strauss A, Corbin JM. Basics of qualitative research: grounded theory procedures and techniques: Sage Publications Inc.; 1990.

[21] Chun Tie Y, Birks M, Francis K (2019). Grounded theory research: a design framework for novice researchers. SAGE Open Med.

[22] McCrae N, Purssell E (2016). Is it really theoretical? A review of sampling in grounded theory studies in nursing journals. J Adv Nurs.

[23] Corbin JM (1998). The Corbin and Strauss chronic illness trajectory model: an update. Sch Inq Nurs Pract.

[24] Nowell LS, Norris JM, White DE, Moules NJ (2017). Thematic analysis: striving to meet the trustworthiness criteria. Int J Qual Methods.

[25] Manuj I, Pohlen TL (2012). A reviewer's guide to the grounded theory methodology in logistics and supply chain management research. Int J Phys Distrib Logist Manag.

[26] Mosavi G. The preparedness of rehabilitation centers in disasters in Zanjan province. Social Welfare Masters Thesis: University of Social Welfare and Rehabilitation Science; 2008.

[27] Dash SR, Mishra US, Mishra P (2013). Emerging issues and opportunities in disaster response supply chain management. Int J Supply Chain Manag.

[28] Jahre M, Heigh I, editors. Does the current constraints in funding promote failure in humanitarian supply chains? Supply Chain Forum: an International Journal; 2008: Taylor & Francis.

[29] Haavisto I, Kovács G, Spens K. Supply chain management for humanitarians: tools for practice: Kogan Page Publishers; 2016.

[30] Mclaren TS, Head MM, Yuan Y (2004). Supply chain management information systems capabilities. An exploratory study of electronics manufacturers. Inf Syst E-business Manag..

[31] Chong AY-L, Liu MJ, Luo J, Keng-Boon O (2015). Predicting RFID adoption in healthcare supply chain from the perspectives of users. Int J Prod Econ.

[32] Bendavid Y, Boeck H (2011). Using RFID to improve hospital supply chain management for high value and consignment items. Proc Comput Sci.

[33] Çakıcı ÖE, Groenevelt H, Seidmann A (2011). Using RFID for the management of pharmaceutical inventory—system optimization and shrinkage control. Decision Support Syst.

[34] Torabizadeh M, Khatami Rad M, Noshadi A (2012). Effect of information system strategies on supply chain strategies and supply chain performance. World Acad Sci, Eng Technol.

[35] Dong S, Xu SX, Zhu KX (2009). Research note—information technology in supply chains: the value of it-enabled resources under competition. Inf Syst Res.

[36] Rachaniotis NP, Dasaklis TK, Pappis CP (2012). A deterministic resource scheduling model in epidemic control: a case study. Eur J Oper Res.

[37] Gupta A, Evans GW, Heragu SS (2013). Simulation and optimization modeling for drive-through mass vaccination—a generalized approach. Simul Model Pract Theory.

[38] Christopher M, Jüttner U (2000). Developing strategic partnerships in the supply chain: a practitioner perspective. Eur J Purchasing Supply Manag.

[39] Ghatari AR, Mehralian G, Zarenezhad F, Rasekh HR (2013). Developing a model for agile supply: an empirical study from Iranian pharmaceutical supply chain. Iran J Pharm Res: IJPR.

[40] Mahendran H, Narasimhan K, Nagarajan N, Gopinath S, editors. Investigation of supply chain risk in the Indian pharmaceutical industry: a case study. Proceedings of the World Congress on Engineering; 2011.

[41] Bazregar R, Khankeh H, Ahmadi S, Hosseini M, Rahgozar M, Moradian M (2013). The evaluation of application of coordination based disaster response model in Rajaye hospital disaster preparedness. Iran J Nurs Res.

[42] Kwon I-W, Kim S-H (2018). Humanitarian supply chain/logistics: roadmap to effective relief effort. J Int Interdiscip Bus Res.

[43] He X, Hu W (2014). Modeling relief demands in an emergency supply chain system under large-scale disasters based on a queuing network. Sci World J.

[44] Ghanbari V, Maddah S, Khankeh H, Karimloo M (2011). The effect of a disaster nursing education program on nurses’ preparedness for responding to probable natural disasters. Iran J Nurs.

[45] Ji G, Zhu C (2012). A study on emergency supply chain and risk based on urgent relief service in disasters. Syst Eng Proc.

[46] Brandeau ML, McCoy JH, Hupert N, Holty J-E, Bravata DM (2009). Recommendations for modeling disaster responses in public health and medicine: a position paper of the Society for Medical Decision Making. Med Decis Making.

